# Radiofrequency Catheter Septal Ablation via a Trans-Atrial Septal Approach Guided by Intracardiac Echocardiography in Hypertrophic Obstructive Cardiomyopathy: One-Year Follow-Up

**DOI:** 10.31083/j.rcm2502038

**Published:** 2024-01-29

**Authors:** Xi Li, Tao Liu, Bo Cui, Yanhong Chen, Cheng Tang, Gang Wu

**Affiliations:** ^1^Department of Cardiology, Renmin Hospital of Wuhan University, Cardiovascular Research Institute, Wuhan University, Hubei Key Laboratory of Cardiology, 430060 Wuhan, Hubei, China; ^2^Department of Cardiology, Wuhan Asia General Hospital, 430060 Wuhan, Hubei, China; ^3^Department of Cardiology, Wuhan Asian Heart Hospital, 430060 Wuhan, Hubei, China

**Keywords:** percutaneous radiofrequency ablation, hypertrophic obstructive cardiomyopathy, transseptal puncture, intracardiac echocardiography

## Abstract

**Background:**

Percutaneous radiofrequency catheter ablation (RFA) in hypertrophic obstructive cardiomyopathy (HOCM) with intracardiac echocardiography (ICE) guidance is a novel method that has been proven to be safe and effective in a small sample size study. RFA of the interventricular septum through a trans-atrial septal approach in HOCM patients with a longer follow-up has not been reported.

**Methods:**

62 consecutive patients from March 2019 to February 2022 were included in this study. The area between the hypertrophied septum and anterior mitral valve (MV) leaflet was established using the three-dimensional system (CARTO 3 system), and all patients received atrial septal puncture under the guidance of intracardiac echocardiography (ICE). Point-by-point ablation was performed to cover the contact area. After ablation, the patients were followed up for 1, 3, 6, and 12 months. Transthoracic echocardiography was performed at 1, 3, 6, and 12 months, and resting and exercise-provoked left ventricular outflow tract (LVOT) gradients were obtained.

**Results:**

During the 1-year follow-up, most patients’ symptoms improved. The NYHA grading of the patient decreased from 2 (2, 3) at baseline to 2 (1, 2) (*p* < 0.001). LVOT peak gradient at rest was decreased from 59 (±27) mmHg to 30 (±24) mmHg (*p* < 0.001), and the provoked peak gradient was decreased from 99 (±33) mmHg to 59 (±34) mmHg (*p* < 0.001). The average maximum septal thickness was reduced from 21 (±4) mm to 19 (±4) mm (*p* < 0.001).

**Conclusions:**

After a 1-year follow-up, ice-guided radiofrequency ablation for HOCM might be a safe, accurate, and effective method. The catheter might be reliably attached to the ablation target area via trans-atrial septal access.

## 1. Introduction

Hypertrophic cardiomyopathy (HCM) is a common heart disease, and more than 75% of HCM patients have left ventricular outflow tract (LVOT) obstruction at rest [1]. Patients with hypertrophic obstructive cardiomyopathy (HOCM) have various symptoms, such as dyspnea, stroke, chest pain, atrial fibrillation, and ventricular arrhythmias, and have significantly higher mortality [2, 3]. In the current guidelines, pharmacological therapy includes non-dihydropyridine calcium channel blockers and β blockers as the first-line treatment [1], but there are still many patients with refractory drug symptoms [4]. The systolic anterior motion (SAM) of the mitral valve (MV) causes the anterior MV leaflet to contact the interventricular septum, which is crucial for the occurrence of severe LVOT obstruction [5]. Accurate and effective septal reduction therapy is essential in relieving the LOVT obstruction. Left ventricular septal myectomy has demonstrated efficacy and safety in septal reduction therapy at high-volume centers of excellence [6]. However, there are patients who would prefer a less invasive procedure. Alcohol septal ablation (ASA) is another acceptable method for patients who are not willing to undergo surgical myectomy or are not suitable for surgical myectomy [1]. However, not all patients have appropriate septal arteries for ASA [7]. Percutaneous radiofrequency catheter ablation (RFA) has been used to treat patients with HOCM [8, 9, 10]. Using intracardiac echocardiography (ICE) technology in RFA has unique advantages. The detailed anatomy of the SAM-septum could be visualized, and catheter tip contact could be monitored by the CARTOR mapping system [11].

The advantages of retrograde aortic or the trans-septal approach for ablation of the interventricular septum have not been reported. Cooper *et al*. [11] reported that retrograde aortic access was more stable than trans-atrial access. We report a larger series of ICE-guided RFA via the trans-atrial septal approach as a septal reduction therapy for HOCM and followed up for 1 year after ablation.

## 2. Methods

From March 2019 to February 2022, patients with resting or exercise-provoked ventricular outflow tract (LVOT) gradient >50 mmHg associated SAM and drug-refractory symptoms were included in this study. All patients were not suited for SM and ASA and received full informed consent, and the local ethical review committees approved this study.

### 2.1 Pre-Procedural

Blood tests, 12-lead electrocardiogram (ECG), transthoracic echocardiogram, cardiac contrast-enhanced CT, 24-hour ECG (Holter), resting and exercise-provoked LVOT gradient, and cardiac function were obtained. Cardiac function was assessed using the New York Heart Association (NYHA) Class classification [12].

### 2.2 General Principles of Ablation Were as Follows

**Fig. 1.**
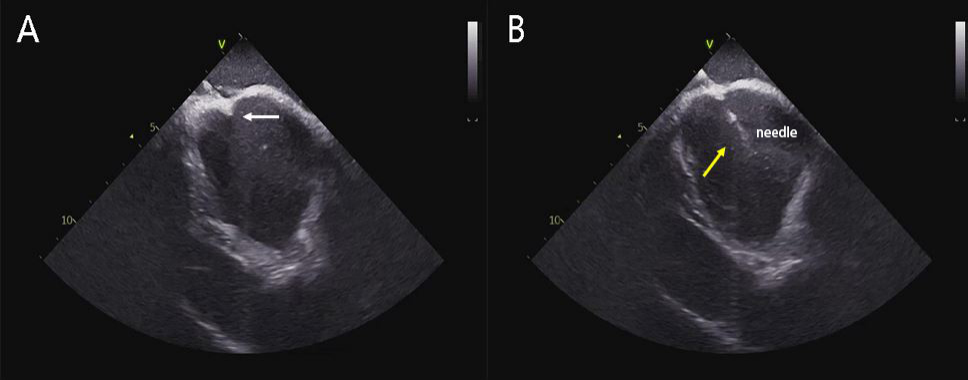
**Trans-septal punctures guided by ICE**. (A) The tip of the transseptal needle falls on the oval fossa, and its “tenting” can be seen (white arrow). (B) The transseptal needle was pushed through the atrial septum, and the drum shadow can be seen. ICE, intracardiac echocardiography.

**Fig. 2.**
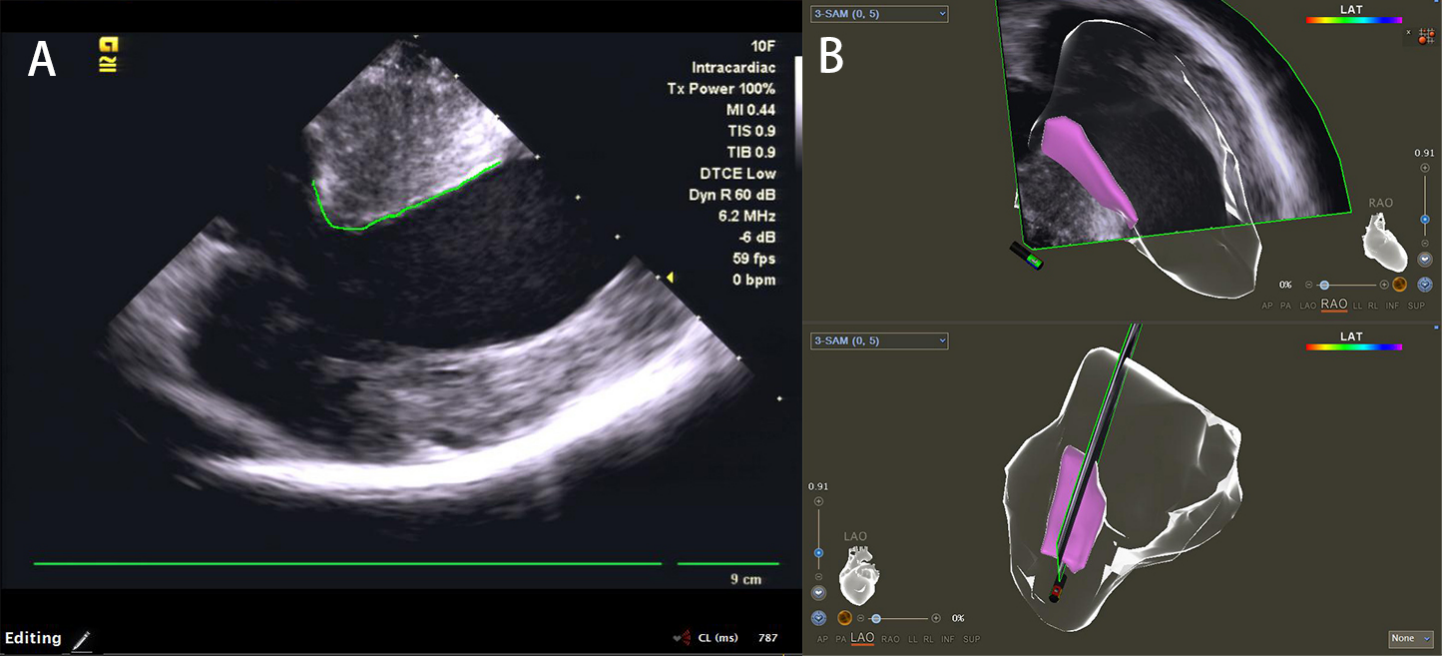
**Ultrasound graphics and three dimensional images of SAM-septal contact area**. (A) SAM-septal contact area of ICE image (green line). (B) SAM-septal contact area in the three dimensional shell (pink area) in RAO and LAO view. ICE, intracardiac echocardiography; SAM, systolic anterior motion; RAO, right anterior oblique; LAO, left anterior oblique; MI, mechanical index; TIS, thermal index in soft tissue; TIB, thermal index for bone; LAT, local activation time.

**Fig. 3.**
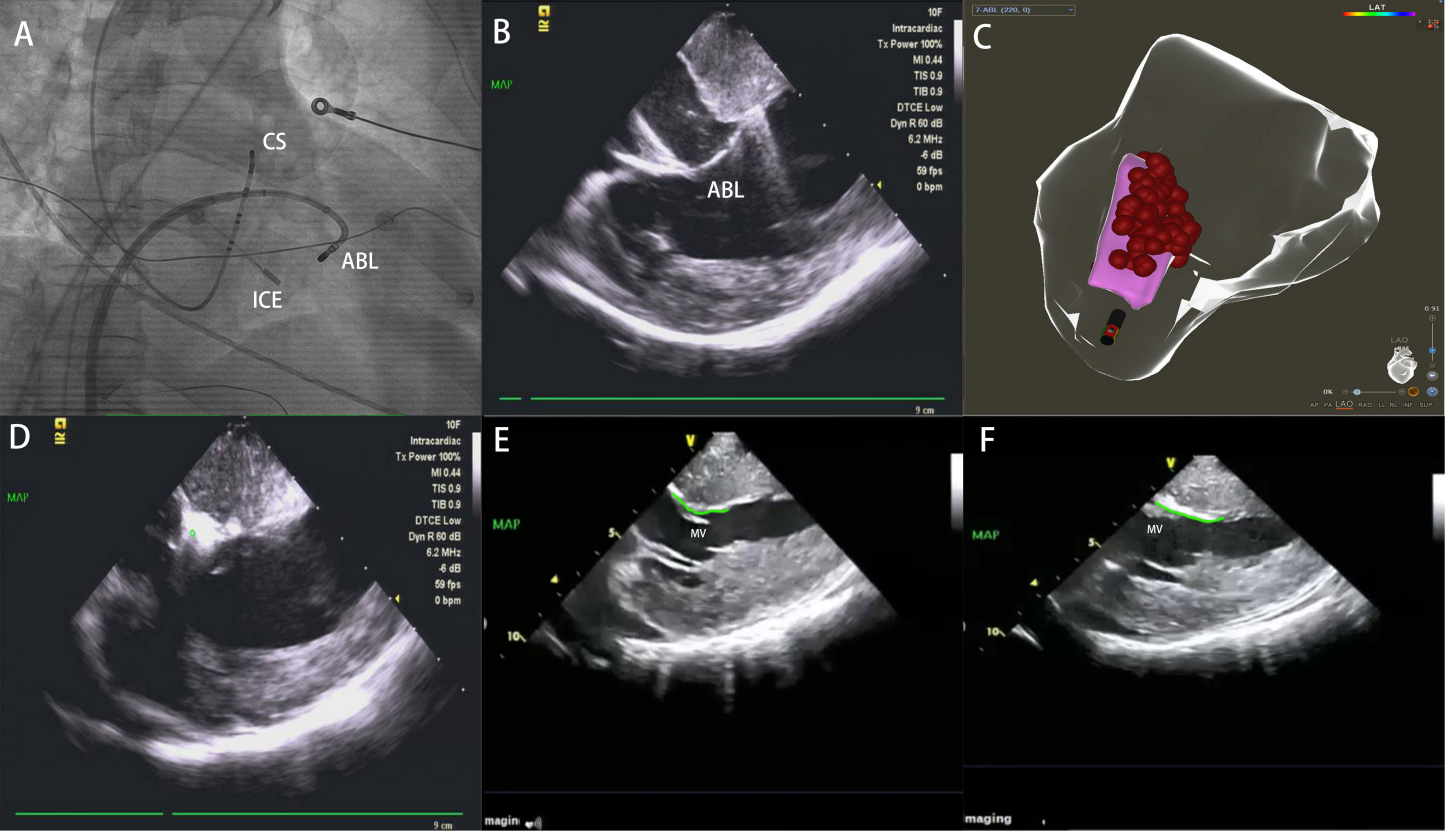
**Ablation at SAM-septal contact area**. (A) The ablation catheter reached the left ventricular septum via the transseptal access. (B) Ablation at left ventricular septum in ICE image. (C) Ablation at SAM-septal contact area in the three dimensional shell (red dots). (D) Edema occurred in the ablation area (Around the green dot). (E) Before ablation of ICE image: Thickened interventricular septum and SAM-septal contact area (green line). (F) After ablation, the interventricular septal thickness was decreased, compared to (E). SAM, systolic anterior motion; ICE, intracardiac echocardiography; ABL, ablation catheter; CS, coronary sinus; MV, mitral valve; MI, mechanical index; TIS, thermal index in soft tissue; TIB, thermal index for bone.

All procedures were performed under general anesthesia. A decapolar coronary sinus (CS) catheter was inserted through the left subclavian vein, and the ${\mathrm{SoundStar}}^{\text{TM}}$ catheter (Biosense Webster, CA, USA) was inserted through the right femoral vein. Then, trans-septal punctures were performed guided by ICE (Fig. 1). Heparin was injected intravenously after the atrial septal puncture, and the activated clotting time (ACT) was maintained >200 s during the procedure. A SAM-septal contact map (regions of contact of the anterior MV leaflet and the hypertrophied septum) was created by ICE images using the CARTO-3 system (Biosense Webster, Diamond Bar, CA, USA) (Fig. 2A,B, **Supplementary Video 1**). Through the trans-atrial septal access, the ablation catheter (ThermoCool Smart Touch STSF; Biosense Webster, CA, USA) was positioned at the LV septum by using a steerable sheath (Agilis, St. Jude Medical, St. Paul, MN, USA). The His bundle was marked before ablation (**Supplementary Fig. 1**). Ablation of the SAM-septal contact area was performed in power control mode (temperature 43 °C; saline irrigation 15 mL/min; power 35–45 W). RF current was delivered for 10–30 s, and the contact force range was 10–15 g (Fig. 3A,B). Procedural endpoints included complete coverage of the SAM-septal contact area and basal septal akinesia [11] (Fig. 3C–F, **Supplementary Videos 2,3**). After ablation, methylprednisolone (80 mg quaque die) was given for 3 days to reduce outflow tract obstruction caused by edema.

### 2.3 Follow Up

The patients were followed up for 1, 3, 6, and 12 months after ablation. An ECG was performed at every visit. Transthoracic echocardiography was performed at 1, 3, 6, and 12 months, and resting and exercise-provoked LVOT gradient, and cardiac function levels were obtained. Patients were strongly recommended to visit a healthcare provider if they felt symptoms.

### 2.4 Statistical Analysis 

The data were expressed as mean ± SD and analyzed by Student’s *t* test. For variables that do not follow the normal distribution, we use the median and interquartile (Q25–Q75) to represent the central trend and variability and compare them with the Mann-Whitney U test. A *p*-value of *p* < 0.05 was statistically significant. All statistical analyses were performed using SPSS (Version 26, IBM Corp, Armonk, NY, USA).

## 3. Results 

A total of 62 consecutive patients were included. Table 1 summarizes the baseline characteristics. The mean age was 56 ± 12 years, and 32 (51.2%) were males. All patients were confirmed to have SAM and outflow tract obstruction by transthoracic echocardiography. All patients had chest pain, chest distress, and/or palpitations. All patients were treated with drug therapy (β-blocker and/or calcium channel blocker) before and after the procedure. 46 (74.2%) patients were in class II ((NYHA), 11 (17.8%) in class III and 5 (8.1%) in class IV. The average septum thickness was 21 (±4) mm. The resting gradient and provoked gradient were 59 (±27) mmHg and 99 (±33) mmHg, respectively. None of the enrolled patients had a history of surgical myectomy (SM) or alcohol septal ablation (ASA). Fifty-seven patients refused SM, and 5 patients could not undergo SM due to poor cardiac function. Fifty-three patients refused ASA, and 9 patients had no ideal septal branch vessel.

**Table 1.** **The baseline characteristics of patients**.

Clinical characteristics		Value
Age (years)		56 ± 12
Male gender, n (%)		32 (51.2%)
Body-mass index, ${\mathrm{kg/m}}^{2}$		25.6 ± 3.5
Hypertension, n (%)		31 (50%)
Diabetes mellitus, n (%)		6 (9.7%)
Coronary artery disease, n (%)		8 (12.9%)
History of stroke or TIA, n (%)		2 (3.2%)
New York Heart Association functional class		
	II	46 (74.2%)
	III	11 (17.8%)
	IV	5 (8.1%)
Drug therapy		
	β-blocker use	36 (58.1%)
	Calcium channel blocker use	42 (67.8%)
Symptoms of HOCM		
	History of syncope	3 (4.83%)
	Chest pain	20 (32.3%)
	Chest distress	37 (59.7%)
	Amaurosis	5 (8.1%)
	Palpitation	20 (32.3%)
Echocardiographic parameters		
	Septum thickness (mm)	21 ± 4
	Resting gradient (mmHg)	59 ± 27
	Provoked gradient (mmHg)	99 ± 33
	SAM, n (%)	62 (100%)
	Outflow tract obstruction, n (%)	62 (100%)

Values are presented as Mean ± SD or as n (%). HOCM, hypertrophic obstructive cardiomyopathy; TIA, transitory ischemic attack; SAM, systolic anterior motion.

### 3.1 Procedure Outcome

The procedural outcome is shown in Table 2. In brief, the total ablation time was 41 ± 28 min. The average ablation area at the left interventricular septum was 2.7 ± 0.6 ${\mathrm{cm}}^{2}$.

**Table 2.** **Procedural data and complication**.

Clinical characteristics		Value
Ablation time (min)		41 ± 28
Power, Watts		46 ± 3
Ablation area of interventricular septum (${\mathrm{cm}}^{2}$)		2.7 ± 0.6
Complication		
	Death, n (%)	0
	Symptomatic stroke, n (%)	0
	Femoral arteriovenous fistula	0
	Femoral pseudoaneurysm	3 (4.8%)
	1st-degree atrioventricular block	1 (1.6%)
	Right bundle branch block	0
	Left bundle branch block	0
	Left anterior fascicular block	0
	Left posterior branch block	6 (9.7%)
	Permanent pacemaker after the procedure	0
	Cardiac tamponade	0

During the 1-year follow-up, there were no deaths, stroke, cardiac tamponade, or major bleeding. One patient had a permanent I atrioventricular block (AVB). Left posterior branch block occurred during the procedures in 8 (12.9%) patients, which recovered in 2 (3.2%) patients but remained permanent in 6 (9.7%) patients during the follow-up. There were no right bundle branch block and left bundle branch block either during ablation or on follow-up. None of the patients required implantation of a permanent pacemaker after ablation. Three patients (4.8%) had femoral pseudoaneurysms, which were successfully eliminated after direct compression.

### 3.2 Follow-Up Outcomes

**Fig. 4.**
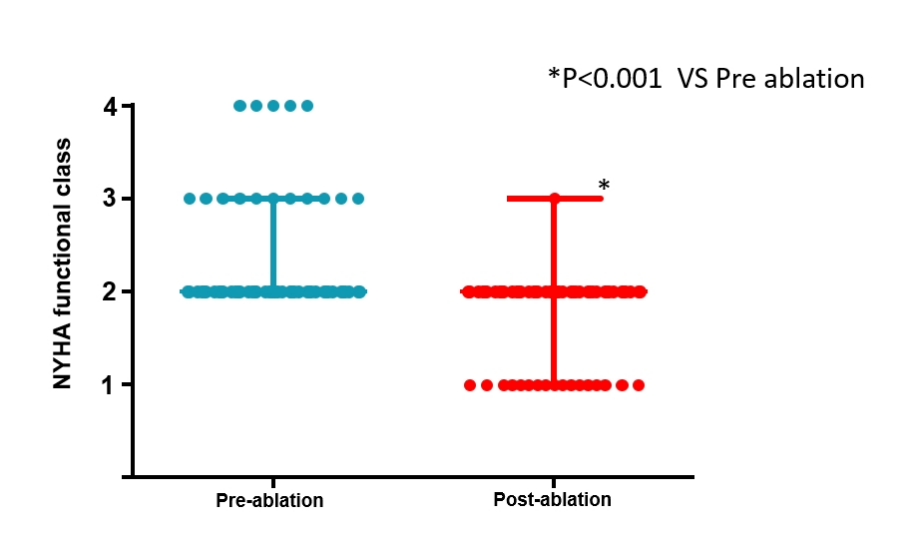
**The changes of NYHA functional class before and after ablation**. NYHA, New York Heart Association. *p < 0.001.

**Fig. 5.**
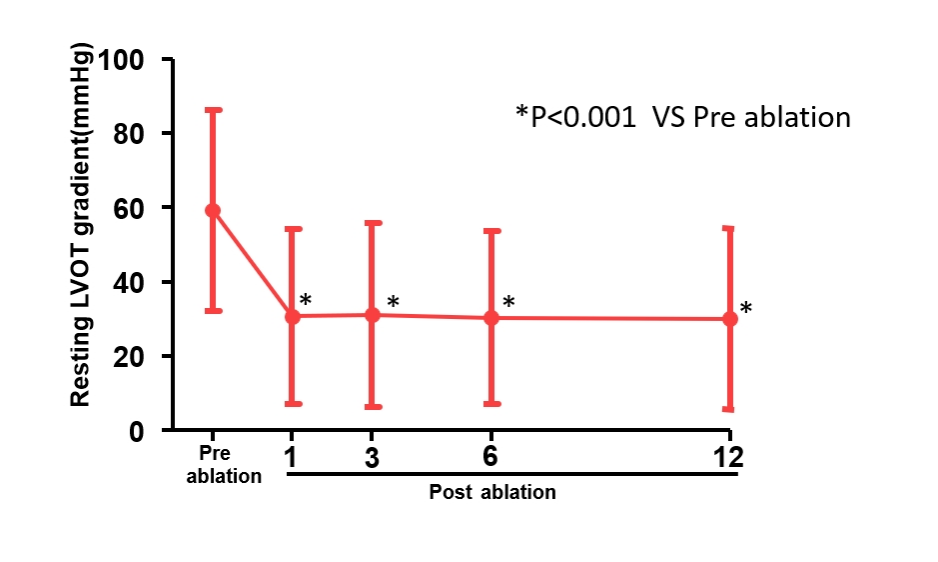
**The peak LVOT gradient at rest pre and post ablation**. The peak LVOT gradient at rest was decreased after ablation (*p < 0.001). The red dotted lines represent a mean value of Pre and post-RFA for resting LVOT gradient. RFA, radiofrequency catheter ablation; LVOT, left ventricular outflow tract.

**Fig. 6.**
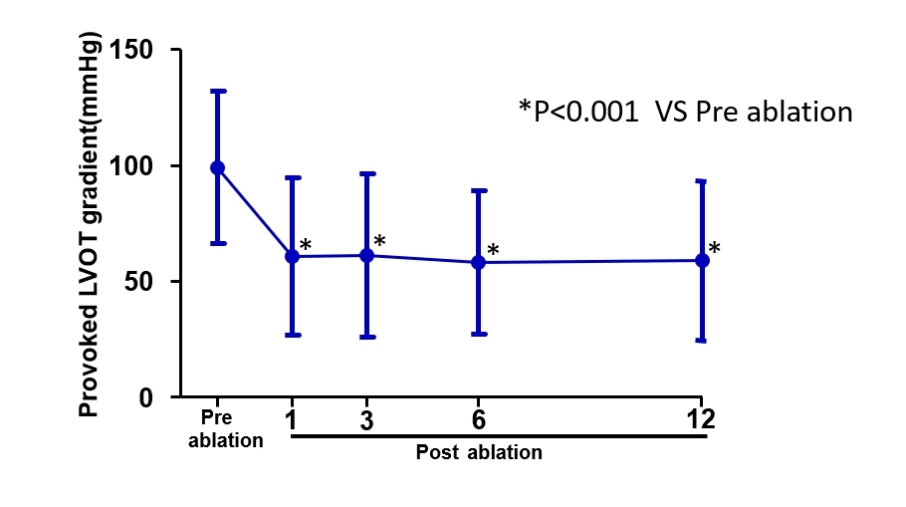
**The provoked peak gradient pre and post ablation**. The provoked peak gradient was decreased after ablation (*p < 0.001). The bule dotted lines represent a mean value of Pre and Post-RFA for provoked LVOT gradient. RFA, radiofrequency catheter ablation; LVOT, left ventricular outflow tract.

During 1, 3, 6, and 12 months of follow-up, most patients’ symptoms improved significantly and had a sustained decreased gradient during 1-year follow-up. At the last follow-up, NYHA class in patients dropped from 2 (2, 3) at baseline to 2 (1, 2) (*p* < 0.001) (Table 3, Fig. 4). The peak LVOT gradient at rest was decreased from 59 (±27) mmHg to 30 (±24) mmHg and provoked peak gradient was decreased from 99 (±33) mmHg to 59 (±34) mmHg (*p* < 0.001) (Table 3, Figs. 5,6). Septal thickness was reduced from 21 (±4) mm to 19 (±4) mm (*p* < 0.001) (Table 3).

**Table 3.** **Clinical outcomes of ablation at last follow-up**.

Clinical results		Pre-ablation	Post-ablation	*p*-value
NYHA functional class		2 (2, 3)	2 (1, 2)	<0.001
Echocardiographic results				
	Septal thickness (mm)	21 ± 4	19 ± 4	<0.001
	LVOTG at rest (mmHg)	59 ± 27	30 ± 24	<0.001
	LVOTG with provocation (mmHg)	99 ± 33	59 ± 34	<0.001

NYHA, New York Heart Association; LVOTG, Left ventricular outflow tract gradient. *p < 0.001.

## 4. Discussion

Our study reported a series of radiofrequency ablations of the interventricular septum for HOCM through a trans-atrial septal approach guided by ICE. Left ventricular septal myectomy can provide near-complete relief of LVOT obstruction and improvement in symptoms with a low mortality rate after the operation [13, 14]. However, good procedural success and low mortality of septal myectomy require high-volume experienced centers. Patients often choose percutaneous procedures if there are other options [15]. Alcohol septal ablation is an alternative to surgical myectomy in HOCM and is a safe procedure with ongoing symptomatic improvement and excellent long-term survival [16]. However, its success relies on suitable septal arterial anatomy and has the risk of procedure-related atrioventricular conduction complications [17]. Radiofrequency (RF) ablation is a new method for septal reduction, which is both minimally invasive and independent of coronary anatomy and has been shown to be feasible in HOCM [18, 19]. The CARTO sound technology in patients undergoing radiofrequency septal ablation defines the ablation target with previously unparalleled accuracy [11]. In our study, we confirmed the effectiveness and safety of this method in a larger patient cohort. The symptoms of most patients were significantly improved, and the incidence of complications was low. In previous studies, RFA in HOCM is effective after 6 months of follow-up [11, 18]. In our study, during a 1-year follow-up, we also confirmed the sustained gradient reduction and symptomatic improvement of this method.

Considering the convenience of this method, whether it can be ablated twice or even many times to further improve the symptoms and gradient reduction of patients will be explored in future studies.

Both trans-atrial septal and retrograde aortic access can reach the left ventricular septum for catheter ablation. Cooper *et al*. [11] tried the 2 methods and preferred the retrograde aortic approach, which was easier to contact the left ventricular septum. In our study, we chose the trans-atrial septal method in all the procedures because the ICE is feasible to guide the trans-septal puncture, and we could detect the optimal puncture site [20, 21]. In this study, we found that under the guidance of ICE and using a steerable sheath, the catheter could be well attached to the interventricular septum of the SAM area by choosing the anterior and lower trans-septal puncture points. Percutaneous arterial cannulation could also increase vascular access site complications [22]. Atrial septal puncture access also reduced arterial vascular access site complications and could shorten the postoperative hospital stay. It is worth noting that during the procedure, we created the SAM-septal contact map by ICE first, then the ablation catheter and steerable sheath crossed the mitral annulus to ablate the SAM-septal contact area by the trans-septal approach. The trans-septal approach crossing the mitral valve with the ablation catheter and steerable sheath might interfere with mitral valve hemodynamics, thereby affecting the definition of the SAM contact area.

Anticoagulant drugs are recommended for at least 1 month after atrial septal punctures to prevent thrombotic events.

During interventricular septal ablation, we usually avoid ablation at the His bundle and left bundle branch area by monitoring the ECG and, at the same time, monitoring the ablation catheter in real-time via intracardiac echocardiography. In our patient cohort, no severe permanent conduction block occurred, and none of the patients required pacemaker implantation post-procedure. Eight patients had left posterior branch block during the operation, and 6 patients had permanent left posterior branch block during the follow-up period. The ECG of 6 patients was followed, and the conduction block did not progress. In order to ensure sufficient ablation area, it may be acceptable to have a left posterior branch block after the procedure in order to avoid the His bundle and left bundle branch trunk area. Previous studies have found that ASA might result in heart rhythm disturbances, especially ventricular arrhythmias (VT) [23, 24, 25]. Catheter ablation at the LV septum may also provide some substrate for VT. ASA might not guarantee complete necrosis of the myocardial tissue around the target vessel, which is the main mechanism leading to ventricular tachycardia. Catheter ablation could accurately ablate the interventricular septum. Real-time monitoring of catheter stability through the ultrasound catheter ensures accurate output of ablation energy, leading to complete myocardial necrosis in the ablation area. No ventricular arrhythmias were found during the follow-up in our study, but a longer follow-up period is needed.

There is no standard ablation energy at present. In our study, we found that ablation energy higher than 40 W is prone to result in steam pop via the trans-atrial septal approach, so we set the upper limit of ablation energy to 40 W. The ablation energy was set at 35–40 W, which continues to ensure the ablation effect while increasing the safety of this technique. However, further clinical research is needed to confirm the optimal ablation energy.

ICE-guided RFA for HOCM was safe, accurate, and effective in our study. However, catheter ablation was limited to the reduction of septal thickness. The interventricular septal thickness decreased from 21 (±4) mm to 19 (±4) mm. Although it was statistically significant, SM or ASA is still the first choice for patients with HOCM.

## 5. Limitations

The main limitation of this study is that it is non-randomized, from a single center, with no long-term follow-up. Prospective randomized controlled studies with larger sample sizes are needed to confirm these findings.

## 6. Conclusions

After a 1-year follow-up, ice-guided radiofrequency ablation for HOCM might be a safe, accurate, and effective method. The catheter might be reliably attached through the trans-atrial septal access during the operation. It was minimally invasive and was an alternative treatment method for those patients who were not suitable for SM or ASA. Further prospective, larger multi-center trials with long-term follow-up are needed to confirm these findings.

## Data Availability

The datasets used and/or analyzed during the current study available from the corresponding author on reasonable request.

## Supplementary Material

Supplementary material associated with this article can be found, in the online version, at https://doi.org/10.31083/j.rcm2502038.
