# Supplementary material for: Radiofrequency Catheter Septal Ablation via a Trans-Atrial Septal Approach Guided by Intracardiac Echocardiography in Hypertrophic Obstructive Cardiomyopathy: One-Year Follow-Up
Source: Rev Cardiovasc Med. 2024 Jan 29;25(2):38. doi: 10.31083/j.rcm2502038 (PMC11263162; doi:10.31083/j.rcm2502038)
Supplement: Supplementary file 1 [file 2153-8174-25-2-038-s1.zip › 2153-8174-25-2-038-s1/Supplementary figure-video.docx]

Supplementary Fig. 1, The region of his bundle was marked with ablation catheter before ablation (yellow dot). His bundle potential (yellow arrow).

Supplementary Video 1, SAM-septal contact map (regions of contact of the anterior MV leaflet and the hypertrophied septum) was created by ICE images using CARTO-3 system.

Supplementary Video 2, ICE image before ablation.

Supplementary Video 3, ICE image after ablation.
