# Supplementary figures and images for: Radiofrequency Catheter Septal Ablation via a Trans-Atrial Septal Approach Guided by Intracardiac Echocardiography in Hypertrophic Obstructive Cardiomyopathy: One-Year Follow-Up
Source: Rev Cardiovasc Med. 2024 Jan 29;25(2):38. doi: 10.31083/j.rcm2502038 (PMC11263162; doi:10.31083/j.rcm2502038)

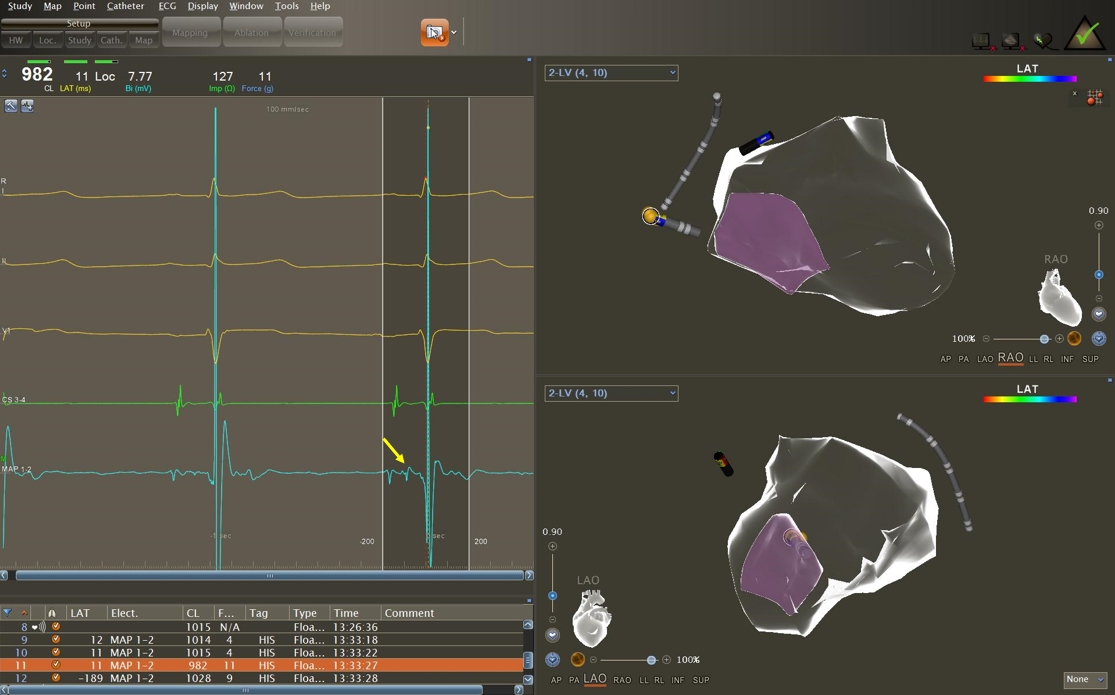

Supplement: Supplementary file 1 [file 2153-8174-25-2-038-s1.zip › 2153-8174-25-2-038-s1/Supplementary Fig. 1.jpg]
